# Effect of Allergen-Specific Immunotherapy on Transcriptomic Changes in Canine Atopic Dermatitis

**DOI:** 10.3390/ijms241411616

**Published:** 2023-07-18

**Authors:** Alicja Majewska, Małgorzata Gajewska, Kourou Dembele

**Affiliations:** 1 Department of Physiological Sciences, Institute of Veterinary Medicine, Warsaw University of Life Sciences (SGGW), Nowoursynowska 159, 02-776 Warsaw, Poland; malgorzata_gajewska@sggw.edu.pl; 2 Department of Small Animal Diseases and Clinic, Institute of Veterinary Medicine, Warsaw University of Life Sciences (SGGW), Nowoursynowska 159, 02-776 Warsaw, Poland; kourou_dembele@sggw.edu.pl

**Keywords:** allergen-specific immunotherapy, canine atopic dermatitis, transcriptome, gene expression, microarrays

## Abstract

Canine atopic dermatitis (cAD) is a genetic, chronic, and recurrent inflammatory and pruritic skin disorder. Allergen-specific immunotherapy (ASIT) is presently recognized as the only clinically effective disease-modifying treatment for allergies. The aim of our study was to analyze the changes in gene expression observed in the peripheral blood nuclear cells of cAD patients subjected to ASIT. Blood samples designated for transcriptomic analyses were collected from AD dogs twice, before and six months after ASIT, and also from healthy dogs. Statistical analysis revealed 521 differentially expressed transcripts, among which 241 transcripts represented genes with well-described functions. Based on the available literature, we chose nine differentially expressed genes (*RARRES2*, *DPP10*, *SLPI*, *PLSCR4*, *MMP9*, *NTSR1*, *CBD103*, *DEFB122*, and *IL36G*) which may be important in the context of the dysregulated immune response observed in cAD patients. The expressions of five out of the nine described genes (*DPP10*, *PLSCR4*, *NTSR1*, *DEFB122*, and *IL36G*) changed after the application of ASIT. The expressions of three of these genes returned to the level observed in the healthy control group. The genes listed above need further investigation to determine details of their role in the molecular mechanism of immune tolerance induction in response to allergen-specific immunotherapy.

## 1. Introduction

Canine atopic dermatitis (cAD) is a genetic, chronic, and recurrent inflammatory and pruritic skin disorder that affects 10% of the canine population. CAD is a complex and multifactorial disease related to immune dysregulation, allergic sensitization, skin barrier defects, microbial colonization, and environmental factors. The inflammatory reaction is caused by an imbalance between the T helper cells Th2 and Th1 and the predominant secretion of the interleukins IL-4, IL-13, IL-5, and IL-31. This induces the recruitment of eosinophils into the inflammatory site and the activation of B lymphocytes, which are stimulated to produce environmental allergen-specific IgE. The binding of the allergen-specific IgE to mast cells causes the degranulation of these cells. The secreted inflammatory mediators along with an insufficient response from T regulatory (Treg) suppressor cells lead to inflammation. Depending on sensitivity to the allergen, the symptoms may be seasonal (e.g., pollen) or not seasonal (e.g., mites). Approximately 80% of dogs with seasonal signs are symptomatic in spring or summer [1,2]. The treatment of atopic dermatitis (AD) is difficult and challenging due to its complex, variable, and multifactorial pathogenesis. The best treatment for AD is avoiding allergens, which is impossible in most cases. For this reason, symptomatic treatment is necessary. The treatment strategy used—either the treatment of acute flares of cAD, the treatment of chronic stages, or the prevention of the recurrence of clinical signs—depends on the clinical condition [3]. Pharmacological treatment is based on the administration of the appropriate drugs, depending on the phase of the disease. Drug treatments may include the topical and/or oral administration of glucocorticoids, ciclosporin, oclacitinib, and lokivetmab. These medicaments treat cAD symptoms, but they can also cause side effects. According to Olivry and Banovic [3], once the patient has remained clear of clinical signs for several weeks, it is time to move to the second phase of AD treatment, described as “proactive therapy”, which aims to prevent the development of flares. At this stage, allergen-specific immunotherapy (ASIT) can be used.

ASIT is presently recognized as the only clinically effective disease-modifying treatment for both human and canine allergy diseases [4,5]. The sensitization method is effective in about 50–75% of dog patients [5,6,7]. ASIT induces long-term clinical tolerance to allergens to which the patient was previously allergic and allows for reduced use of systemic anti-inflammatory agents, thus improving the quality of life of patients suffering from AD. Despite the positive effects of treatment, in most cases, the exact molecular mechanisms of ASIT are still not completely elucidated.

The induction of immune tolerance to an allergen through ASIT is a complex process which involves both innate and adaptive immunity. The influence of innate immunity is mainly based on the inhibition of the activation and degranulation of effector cells, such as basophils and mast cells, as well as the modulation of the action of dendritic cells and decreasing the level of type 2 innate lymphoid cells [8,9]. The latter, owing to the production of IL-5 and IL-13, are involved in allergic inflammation [9]. Changes in adaptive immunity are related to the induction of allergen-specific T regulatory (Treg) and B regulatory (Breg) cells, which produce the crucial cytokines (IL-10, TGF-β and IL-35) for suppressing allergic inflammation. The suppressive milieu causes a decline in IgE production and induces the production of IgG4 by Breg cells. The IgG4 antibodies compete with IgE for the allergen, neutralize the allergen, and block the formation of the allergen IgE complex, therefore inhibiting the degranulation of effector cells (basophils, mast cells, and eosinophils). The suppressive regulatory cells cause a decrease in the number and activity of Th2 cells, involving a shift from a Th2 to a Th1 immune response, and modulate the activity of dendritic cells [9,10,11,12].

Much more is known about the abovementioned cellular mechanisms of the response to ASIT in humans as this has been extensively studied. Much less is known in the case of cAD. There have been very few studies referring to the use of ASIT in dogs, and the results described in these reports are variable. Most of them have focused on the effectiveness of the therapy and its ability to alleviate clinical signs and prevent the progression of the disease, leaving the mechanism of the immune response poorly described. There are several reports on changes in the level of cytokines involved in the immune response or in the number of Treg cells. Often, these results are ambiguous and not easy to interpret. There are also no reports on whether and how immunotherapy affects the regulation of gene expression. Therefore, the aim of our study was to analyze the changes in the gene expression of peripheral blood nuclear cells (PBNCs) observed in cAD patients subjected to ASIT. The choice of tissue analyzed in this research was not random, but based on the fact that blood samples constitute a valuable source of information about the state of an organism and are easier to collect than skin samples. The use of the microarray technique enabled us to seek genes that are differentially expressed in healthy dogs and cAD patients before and after ASIT. The results of our study identify several new candidate gene-encoding proteins that are directly or indirectly involved in signaling pathways that are important for the induction of immune tolerance to allergens. The determination of new potential molecular markers of cAD showing differential expression in the PBNCs of dogs affected with this disease and of those subjected to ASIT may be helpful in finding new therapeutic targets in the future.

## 2. Results

**Figure 1:**
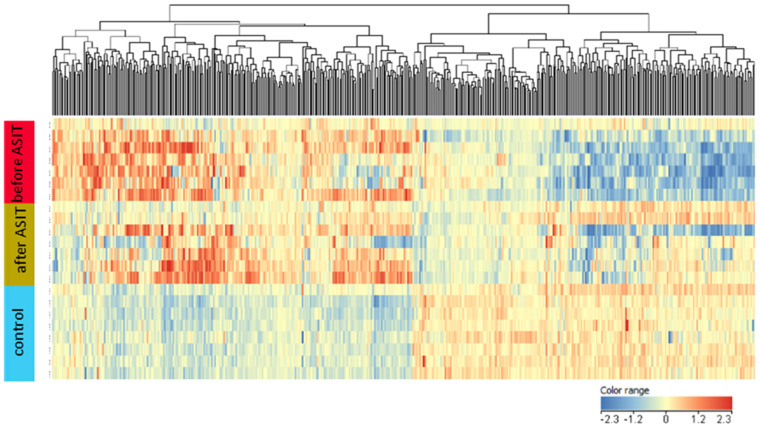
Heat map presenting supervised hierarchical clustering of differentially expressed genes for three investigated groups (healthy control, and cAD patients before ASIT and 6 mounts after ASIT). The distances were calculated using the Euclidean correlation metric and Ward’s method. The columns show individual genes, and rows represent individual patients. Red color indicates increased gene expression, whereas blue color indicates decreased expression of genes.

**Figure 2:**
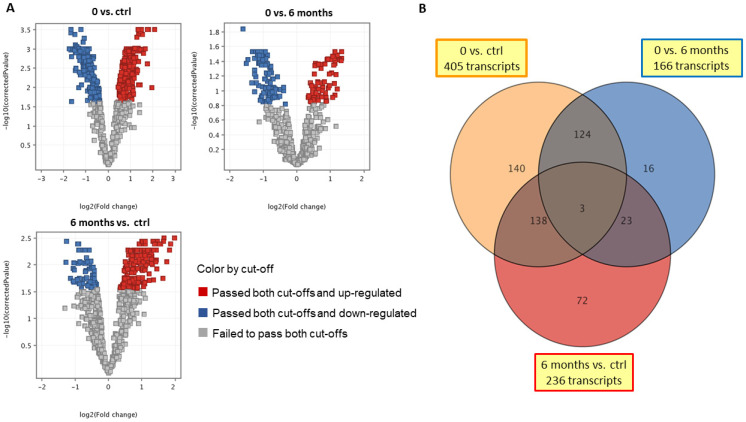
(**A**) Volcano plots presenting relationship between corrected *p* value (log10) and fold change (log2) in genes that were differentially expressed in cAD patients before therapy in comparison to healthy control group (0 vs. ctrl), in cAD patients before therapy in comparison to patients 6 months after ASIT (0 vs. 6 months), and in cAD patients 6 months after ASIT compared to healthy control group (6 months vs. ctrl). (**B**) Venn diagram based on microarray analysis showing number of differentially expressed genes in tested groups: cAD patients before therapy vs. healthy control group (0 vs. ctrl), cAD patients before therapy vs. 6 months after ASIT (0 vs. 6 months), and cAD patients 6 months after ASIT vs. healthy control group (6 months vs. ctrl). The above results were obtained using one-way ANOVA and Tukey’s HSD post hoc test (*p* < 0.05) and Benjamini and Hochberg multiple testing correction (FDR < 0.05).

In this study, gene expression in PBNCs was tested using a microarray technique in AD dogs subjected to ASIT for 6 months. Six out of seven dogs receiving ASIT showed a positive response to the therapy. A detailed description of the patients’ responses to the ASIT and the changes in their cytokine profiles and lymphocyte subpopulations is presented in our previous publication [13]. The microarray analysis was performed to search for differentially expressed genes that can be detected in the blood samples of cAD patients before ASIT and after 6 months of treatment. In this transcriptomic analysis, differentially expressed genes in PBNCs were compered among three groups of dogs: healthy dogs—control (ctrl), dogs with AD before treatment (0), and dogs with AD after 6 months of ASIT (6 months). The following comparisons were analyzed: dogs with AD before treatment vs. healthy control dogs (0 vs. ctrl); dogs with AD after 6 months of ASIT vs. healthy control dogs (6 months vs. ctrl); cAD patients before and after 6 months of ASIT (0 vs. 6 months). Statistical analysis revealed 521 differentially expressed (DE) transcripts. Out of these probes, it was possible to identify 241 transcripts representing specific genes. The profiles of the differentially expressed genes of the three investigated groups of dogs are presented on a heatmap (Figure 1). The expressions of 405 transcripts in dogs with AD and 236 transcripts in dogs after 6 months of treatment were regulated relative to the healthy control (Figure 2). Within this group of transcripts, 141 DE genes were common in both comparisons (0 vs. ctrl, and 6 months vs. ctrl); whereas ASIT caused changes in the expression of 166 genes compared to before treatment (6 months vs. 0) (Figure 2). Table 1 presents differentially expressed genes that we found interesting in the context of canine atopic dermatitis and applied ASIT. The expressions of some of these genes have never been associated with atopic dermatitis, nor have they been analyzed in cAD. A full list of significantly differentially expressed genes is presented in the Supplementary Materials (Table S1).

**Figure 3:**
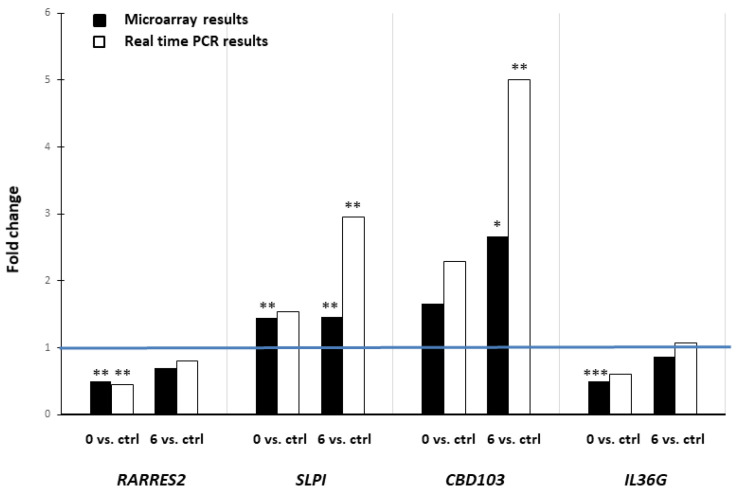
Expression of *RARRES2*, *SLPI*, *CBD103*, and *IL36G* genes in peripheral blood nuclear cells of cAD patients before ASIT and 6 months after ASIT and in healthy control dogs. Gene expression was analyzed using microarray and real-time PCR. Data are presented as fold change in gene expression in cAD patients before ASIT vs. healthy control dogs (0 vs. ctrl) and in cAD patients 6 months after ASIT vs. healthy control dogs (6 vs. ctrl). Bar graph presents upregulated genes with fold change values >1, and downregulated genes with fold change values <1. Statistically significant differences are depicted as * *p* < 0.05, ** *p* < 0.01, *** *p* < 0.001.

The microarray data were validated using real-time quantitative PCR to confirm changes in the gene expression obtained from microarray analysis. The expressions of four selected genes, *CBD103*, *IL36G*, *RARRES2*, and *SLPI*, were analyzed (Figure 3).

## 3. Discussion

The molecular mechanisms of ASIT in cAD treatment have not been fully elucidated yet. Furthermore, existing reports describing the immune response of AD dogs subjected to ASIT often show incoherent results. For example, a study by Hou and coworkers [14] demonstrated an increase in total IgG antibodies in response to various antigens from mites after using ASIT in dogs. Research published later by DeBoer and coworkers [15] showed that after 6 months of using sublingual immunotherapy (SLIT), serum mite-specific IgE dropped, and mite-specific IgG levels increased, but were more variable over time. In addition, the changes described did not completely reflect the clinical response, and were not always associated with clinical improvement [15]. In a study by Keppel and coworkers [16], an increase in the concentration of IL-10 was reported after ASIT, but recently, another research group found no difference in the level of IL-10 between healthy and AD dogs after using different combinations of ASIT therapies [17]. Only intralymphatic immunotherapy (ILIT) caused changes in the level of IL-10 [17]. A previous study by our research group showed that only in some cAD patients after 6 months of ASIT did the level of IL-10 increase [13]. The data regarding the role of Treg suppressor cells are also inconsistent. Some studies have shown that the number of Treg cells is the same in healthy and atopic dogs, and increases after therapy [16], whereas other reports describe a higher number of Treg cells in cAD patients than in healthy dogs [18,19]. Our research [13,20] showed more Treg cells in atopic than in healthy dogs. After 3 months of ASIT, the number of Treg cells decreased, but after 6 months it increased again. In addition, our studies showed a decrease in IL-13 levels during ASIT, suggesting a shift in the immune response from Th2 to Th1. In parallel, we observed a decrease in the level of TNF-α and in the number of CD8^+^ cells, which may be linked with a reduced inflammatory response [13]. It should be added that six out of seven dogs receiving ASIT showed a positive response to the therapy [13]. Therefore, in the continuation of our research, we focused on the possible use of peripheral blood nuclear cells as a source of information about the changes in gene expression that could be characteristic of cAD and could be regulated by ASIT. The results obtained demonstrated a substantial number of differentially expressed genes. When analyzing the data from the microarray experiment, we focused on several genes that were regulated in the three compared groups of dogs (healthy control, AD dogs before ASIT, and AD dogs 6 months after ASIT) (Table 1). These genes have not been previously described in the literature as markers of atopic dermatitis, but their function seems to be directly or indirectly associated with cAD.

One of the differentially expressed genes found in our study is *retinoic acid receptor responder protein 2 (RARRES2)*, also known as *TIG-2 (tazarotene-induced gene-2)*. *RARRES2* showed significantly lower expression in AD dogs than in healthy control dogs (0 vs. ctrl), and also lower expression (although not significantly) in dogs after six months of ASIT (6 months vs. ctrl; 0 vs. 6 months). This gene encodes prochemerin, which can be converted into an active form of chemokine known as chemerin. Chemerin binds to the retinoic acid receptor, causing chemoattraction of dendritic cells and macrophages, which links innate and adaptive immunity [21,22]. *RARRES2* belongs to the group of retinoid response target genes. Several skin diseases, including AD and psoriasis, are related to alterations in retinoid metabolism/signaling [23,24]. Mihály and coworkers [24] showed that the all-trans retinoic acid (ATRA) concentration was lower in both lesional and non-lesional skin in human AD patients. The expression of the *RARRES2* gene was downregulated in AD skin in comparison with skin from healthy volunteers [20]. Although there are no available data referring to the changes in *RARRES2* expression in peripheral blood cells, or the molecular mechanism of chemerin activity in relation to AD, a protective role of the chemokine in other allergic diseases, such as allergic asthma, has been documented. A study using a BALB/c mouse model demonstrated that the administration of chemerin attenuated allergic airway inflammation, decreased the accumulation of CD4^+^ T cells and eosinophils in the bronchoalveolar lavage fluid of mice with allergic asthma, and decreased the gene expression of the Th2-attracting chemokines CCL17 and CCL22, causing suppression of the airway recruitment of inflammatory CD11c^+^CD11b^+^ dendritic cells [22]. These results suggest that *RARRES2* expression is downregulated in various allergic diseases, and perhaps the use of chemerin could have a positive protective or therapeutic effect.

Another gene that showed significantly lower expression in the cAD group compared to the healthy control (0 vs. ctrl) and the treated group of dogs (0 vs. 6 months) was *DPP10 (dipeptidylpeptidase-like 10)*. The expression of the *DPP10* gene can be connected to the expression of the *SLPI (secretory leukocyte peptidase inhibitor)* gene, which showed the same level in cAD patients before and after therapy (0 vs. 6 months), and was significantly upregulated compared to the healthy control dogs (0 vs. ctrl; 6 months vs. ctrl). These genes have not been previously described in the context of AD, but some studies link the expression of *DPP10* and *SLPI* with allergy symptoms. According to Zhang and coworkers [25], point mutation in the *DPP10* gene leads to increased airway responsiveness following allergen challenge. The overexpression of *DPP10* significantly enhanced glucocorticoid receptor (GR) activation even without glucocorticoid treatment. This suggests that the DPP10 protein may influence endogenous anti-inflammatory corticosteroid production. Meanwhile, the knockdown of *DPP10* in human airway epithelial cells diminished the ability of GR to translocate to the nucleus and bind to DNA, and the overexpression of *DPP10* caused a reduction in the level of SLPI induced by IL-1β. TNF-α and IL-1β act in synergy, but also, TNF-α stimulates the secretion of IL-1β. In our studies, we did not test the level of IL-1β; however, we determined the level of TNF-α in the plasma of the investigated dogs [13]. The highest concertation of TNF-α in the plasma was noted in cAD patients before therapy. Perhaps TNF-α could have complicity in the upregulation of *SLPI* expression. The human *DPP10* gene was identified as a candidate gene for the prognosis of susceptibility to asthma, a common disease of the airways involving atopic inflammation and hyper-responsiveness to various agents [25,26]. This suggests that DPP10 may also play a protective role in cAD. On the other hand, the SLPI protein was shown to mediate the suppression of TGF-β expression and interfere with the differentiation of Treg cells [27]. Additional elastase activity is needed for the higher level of TGF-β expression in dendritic cells, which promotes an increase in the number of CD4^+^FOXP3^+^ cells [28].

Our study also determined that the *PLSCR4 (phospholipid scramblase 4)* gene showed differential expression, with the highest expression occurring in AD dogs before therapy (0 vs. ctrl; 0 vs. 6 months), whereas its expression in the healthy control dogs and in the cAD patients after ASIT was similar (6 months vs. ctrl). Py and coworkers [29] demonstrated that the phospholipid scramblase membrane proteins (PLSCR1 and PLSCR4), which largely localize in the membranes of T lymphocytes CD4^+^, are receptors for SLPI, but also interact with the CD4 receptor. The SLPI protein can disrupt the association between PLSCR1 and CD4. SLPI binding to the endofacial domain of PLSCR1 and PLSCR4 can induce its translocation from the extracellular matrix to the cytoplasm and nuclei of monocytes, macrophages, and B and Th lymphocytes. Perhaps the increased expression of the *SLPI* gene is connected with the increased expression of the *PLSCR4* gene observed in our study.

Our transcriptomic analysis also demonstrated changes in the expression of the *MMP9* gene *(encoding matrix metalloproteinase 9)* among the groups of dogs subjected to this study. *MMP9* expression was similar in two cAD groups (before and after ASIT (0 vs. 6 months)) and significantly higher compared to the healthy control group (0 vs. ctrl; 6 months vs. ctrl). MMP-9 (also known as gelatinase B) and other metalloproteinases are able to degrade extracellular matrix (ECM), enabling tissue remodeling and cell migration. MMP-9 is one of the key enzymes in the development and course of the inflammatory reaction following allergen challenge. Several inflammatory cells secrete MMP-9, such as eosinophils, neutrophils, T cells, macrophages, and mast cells. This metalloproteinase promotes the migration and activation of immune cells by cleaving pro-inflammatory chemokines and cytokines, and therefore, contributes to the inflammatory processes [30,31,32]. Zhang and coworkers [33] indicated the dependence of the migration of CD4+ cells on MMP activity. The migratory capacity of Th1 cells was higher than that of Th2 cells, and there was also a difference in the levels of metalloproteinases (MMP-2 and MMP-9) secreted by the Th1 and Th2 cells. A similar effect was observed in human and murine cells [34]. Harper and coworkers [35] indicated the dominant presence of MMP-8 and MMP-9 in a mixture of MMPs detected on the skin surface in acute human AD. The presence of MMP-9 in this environment is associated with the presence of immune cells, such as eosinophils, lymphocytes, and dendritic cells, which require MMP-9 activity to penetrate the inflamed skin. The expression of the *MMP-9* gene was elevated in acute compared with chronic AD lesions. Purwar and coworkers [36] demonstrated the influence of IL-13 on MMP-9 expression in the basal keratinocyte layer of human skin biopsies. The addition of TNF-α to human peripheral blood eosinophils cultured in vitro from atopic asthmatic patients, stimulated a 95% increase in MMP-9 activity above baseline [37]. Our results showed a significant increase in the levels of IL-13 and TNF-α in the blood of cAD patients before therapy [13]. It is possible that these cytokines are among the factors contributing to the increase in *MMP9* gene expression.

Furthermore, our present study demonstrated higher expression of both *SLPI* and *MMP9* genes in AD dogs. The SLPI protein was shown to have a regulatory effect on MMP-9 quantity through transcriptional upregulation. SLPI may either directly or indirectly induce the transcription of *MMP-9*. This, coupled with SLPI extracellular interaction with plasmin, regulates MMP-9 activation and release. Thus, the net pro-invasive effect of SLPI on MMP-9 results from both increased gene transcription and protein production and secretion, tempered, in part, by SLPI’s inhibition of the plasmin activity required to cleave the propeptide from MMP-9 [38]. A study by Poachanukoon and coworkers [39] showed, additionally, that active proteases present in allergen extract from house dust mites (HDM) directly activate MMP-9 by cleaving pro-MMP-9. It can be assumed that even though the activity of MMP-9 comprises SLPI’s inhibition of plasmin, this metalloproteinase can be activated by HDM or other allergen proteinases in allergy patients.

Another differentially expressed gene that could potentially be important in cAD is *NTSR1 (neurotensin receptor 1*, also known as *NTR*). Significantly, the highest expression of *NTSR1* was noted in cAD patients before ASIT compared to the healthy control dogs (0 vs. ctrl) and to cAD patients after therapy (0 vs. 6 months). The lowest expression was observed in the cAD group after therapy, but the difference was not significant (6 months vs. ctrl). The NTSR1protein induces intracellular signaling through phospholipase C and the inositol phosphate signaling pathways. It also functions through the production of cGMP, cAMP, and arachidonic acid, through the MAP kinase pathways, and through the inhibition of Akt activity [40]. Neurotensin (NTS), which is a ligand for NTSR1, functions as a neurotransmitter in the nervous system, and as a hormone in the peripheral tissues. Its action in the periphery is mediated by the G protein-coupled receptor, NTSR1 [41,42]. Aside from the role of neurotensin in the nervous system, it is also known for its pro-inflammatory role, induction of vasodilatation, vascular permeability, activation of mast cell degranulation, and enhancement of the directional migration and phagocytosis of neutrophils [43]. NTS can be involved in the pathogenesis of inflammatory skin disorders, including AD, especially those exacerbated by stress, scratching, and sweating [44], however the mechanism is still not well understood. It was shown that NTS concentration increased in rodent skin as a consequence of acute stress, and induced vascular permeability acting on mast cells through NTS receptors expressed by these cells [45,46]. Some studies indicated [47,48] that NTS also stimulated rodent mast cells to secrete histamine through NTSR. Alysandratos and coworkers [49] demonstrated a relationship between the corticotropin-releasing hormone (CRH) and neurotensin (both released in the stress response) and their receptors (CRHR-1 and NTSR) in a human culture of mast cells. CRH induced *NTS* and *NTSR1* gene expression, whereas NTS induced *CRHR1* gene expression. The authors also showed that NTS stimulated human mast cell degranulation and the release of VEGF, which contributed to skin vascular permeability, and intensified the effect of CRH on VEGF release through NTSR. An increased level of VEGF was observed in the serum of AD patients, and this growth factor can be synthesized and released by different cells (platelets, eosinophils, and mast cells) [50,51]. Studies also demonstrated that the level of NTS in the serum of AD patients was higher than in that of control patients, similarly to the expression of *NTS* gene, which was increased in the lesional skin of AD patients in comparison to controls [52]. However, there was no difference in the expression of the *NTSR1* gene in the lesional skin of AD patients compared to the control [52]. NTS and NTSR proteins were detected in the lesional skin, but not in the skin of healthy control individuals. Additionally, NTSR activation also results in the secretion of the inflammatory cytokines IL-8 and TNF-α [49]. Our previous study demonstrated an increased level of TNF-α in the plasma of cAD patients before treatment [13], and the microarray analysis revealed an elevated expression of the *NTSR1* gene in AD dogs before treatment. In the available literature, there is no information about the role of neurotensin and its receptor (NTSR1) in mast cells localized in the canine skin or in the peripheral blood, and there is no published research on the role of these proteins in the course of cAD. However, it seems that this neuropeptide and its receptor should be further investigated in the context of cAD.

*CBD103* and *DEFB122* were the next interesting genes to show differences in expression. Both genes encode proteins belonging to β-defensins: *beta-defensin 103 (CBD103)* and *beta-defensin 122 (DEFB122)*. The highest expression of *CBD103* was noted in the dogs after ASIT and differed significantly compared to the expression detected in the healthy control dogs (6 months vs. ctrl). CAD patients before therapy also showed a higher expression of the *CBD103* gene compared to the healthy control dogs (0 vs. ctrl), but this difference was not statistically significant. However, the expression of *DEFB122* was significantly lower in cAD patients before therapy compared to the healthy control dogs (0 vs. ctrl) and AD dogs after ASIT (0 vs. 6 months). It seems that immunotherapy increased the expression of *CBD103*, but the mechanism of this process remains unclear. In the case of *DEFB122*, immunotherapy restored the expression of this gene to a normal level. In the cAD group, before immunotherapy, the expression of *DEFB122* was reduced. It is difficult to interpret the observed changes in the expression of these two defensins. β-defensins play a role in both innate and adaptive immunity, and are considered a bridge between the innate response and adaptive immune cell requirements [53,54]. β-defensins are cationic peptides, which show antibacterial activity. These proteins can directly kill microbial pathogens through their interaction with anionic components in the microbial membrane [55,56]. Canine atopic dermatitis is accompanied by secondary microbial infections most often caused by *Staphylococcal pyoderma* and *Malassezia dermatitis* [1]. It is possible that abnormal levels of defensins may contribute to a defective innate immune response. Beta-defensins may show pro- and anti-inflammatory effects that depend on many factors, including the disease stage and exposure to pathogens, among others [53]. Beta-defensins can affect the chemoattraction of CD4+ memory T cells and immature dendritic cells by binding to CCR6 [57], but these proteins can also suppress the effects of TNF-α and IL-6.

Van Damme and coworkers [56] detected the expression of the *CBD103* gene in both the skin and peripheral blood mononuclear cells of all tested healthy dogs, as well as in many other tissues (duodenum, kidneys, testes, bone marrow, and lungs), whereas *DEFB122* was not detected in all dogs. *DEFB122* was expressed in over 50% of skin samples from the tested dogs, but not in the peripheral blood. In a study by Lancto and coworkers [58] the expressions of the *CBD103* and *DEFB122* genes were reported in skin samples from various body sites of healthy dogs (axilla, forehead, inner thigh, scapula, and ventral abdomen). Van Damme and coworkers [56], as well as Lancto and coworkers [58], showed decreased expression of the *CBD103* gene in the skin of AD dogs with and without lesions compared to the skin of healthy dogs. On the other hand, Leonard and coworkers [59] reported no difference. All authors showed no difference in *CBD103* expression between lesional and non-lesional skin in cAD patients. Lancto and coworkers [58] indicated a lower level of the *DEFB122* transcript in the lesional and non-lesional skin of AD dogs than in the skin of healthy dogs. The results of the present study, together with the reports of other researchers, highlight the need for further research into the role of β-defensins (CBD103 and DEFB122) in cAD.

Our analysis of the microarray results also showed a significant reduction in *IL-36γ* expression in dogs with AD before therapy compared to the healthy control dogs (0 vs. ctrl) and AD dogs after ASIT (0 vs. 6 months). IL- 36γ is one of the IL-36 isoforms belonging to the IL-1 superfamily, acting as a receptor agonist for pro-inflammatory functions. IL-36R ligands bind to the IL1RL2/IL-36R receptor and use the IL-1 receptor accessory protein (IL-1RAcP) as a co-receptor that triggers signal transduction, and activates mitogen-activated protein kinase (MAPK), and nuclear factor-kappa B (NF-kB) signaling pathway [60]. IL-36 is involved in immune cell activation, antigen presentation, and pro-inflammatory factor production [61]. The IL-36 interleukins are most active in barrier tissues, like the skin, lungs, bronchia, and intestines. Their main function is to regulate the interactions of the environment and the body, because they serve as the first line of defense against microorganisms [62]. IL-36 expression can be found in keratinocytes, B-lymphocytes, T-lymphocytes, dendritic cells, and monocytes [63,64,65,66,67,68]. In normal skin, IL-36 cytokines are expressed constitutively at low levels. Jiang and coworkers [69] determined that IL-36γ is important for the control of wound healing after skin injury. mRNA expression and the protein abundance of IL-36γ were elevated in wounded skin. However, the overexpression of IL-36γ appears to be problematic, as it may cause the loss of homeostatic balance, which leads to a pathological pro-inflammatory milieu and to disorders such as psoriasis. Many authors suggest that IL-36 may be a biomarker of psoriasis, and is also used as a therapeutic target. However, the level of *IL-36γ* expression in other inflammatory skin diseases, such as atopic dermatitis, lichen planus, contact eczema, subacute cutaneous lupus erythematosus, and mycosis fungoides, was found to be much lower than in psoriasis [67]. Genes with higher expression in atopic dermatitis are induced to a greater extent by the Th2 cytokines IL-13 and IL-4 than by the cytokines IL-17A, IL-17A/TNF, IL-36α, β, γ, and IFN-α; thus, the low expression of the *IL-36γ* gene observed in our study in the PBNCs of cAD patients is in agreement with this theory [70]. In a study by D’Erme and coworkers [71], the level of the *IL-36γ* transcript in AD skin was slightly higher than in healthy skin, but these results were not statistically significant. Our immunohistological analyses demonstrated a very low level of IL-36 protein in atopic skin. Also, other authors found no changes in *IL-36γ* expression in the eczematous skin of atopic dermatitis patients compared to the healthy control [72]. Other studies have shown that *IL-36* expression depends on the phase and severity of the disease. Increased expression of *IL-36α*, *IL-36γ*, and *IL-36 Ra* was demonstrated in the lesional skin of AD patients compared to non-lesional skin [67,73]. Tengvall and coworkers [74] reported increased expression of *IL-36γ* in cAD skin with mild lesions versus healthy control skin. It should be emphasized that all the results described above refer to the changes in *IL-36γ* expression directly in skin samples, whereas in our study, the lower expression of the *IL-36γ* transcript was characteristic of nuclear blood cells of dogs with AD. Perhaps the deficiency of IL-36γ causes disorders in the maintenance of homeostasis, which is important in the first line of defense and wound healing. The decreased expression of the *IL-36γ* gene observed in our study in the PBNCs of cAD patients may be one of the factors contributing to the overreaction to the allergen, and should be further investigated as a potential marker in this disease. 

**Figure 4:**
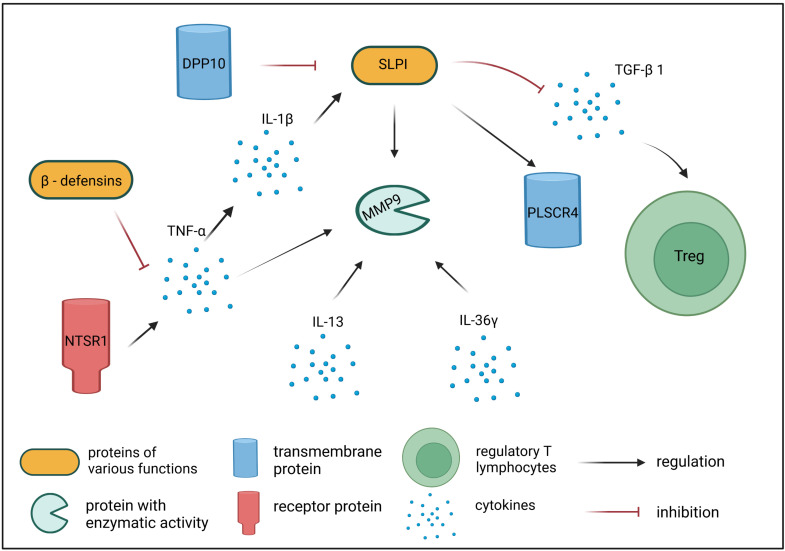
Scheme of potential direct and indirect interactions among proteins encoded by differentially expressed genes detected in the microarray analysis, and cytokines linked with the course of cAD and ASIT used for cAD treatment. Scheme was created using BioRender.com.

**In summary**, the present study analyzed changes in the transcriptomic profiles detected in the PBNCs of dogs affected by AD in comparison to healthy dogs and in comparison to their state after 6 months of conducting ASIT. Statistical analysis revealed 521 differentially expressed transcripts, among which 241 transcripts represented genes with well-described functions. Based on the available literature, we chose nine differentially expressed genes (*RARRES2*, *DPP10*, *SLPI*, *PLSCR4*, *MMP9*, *NTSR1*, *CBD103*, *DEFB122*, and *IL36G*) that may be important in the context of dysregulated immune responses observed in cAD patients. Table 2 summarizes the functions of the genes described. Eight of these nine genes (except *CBD103*) showed different expression levels in cAD patients before therapy compared to healthy dogs. The expression of five of the nine described genes (*DPP10*, *PLSCR4*, *NTSR1*, *DEFB122*, and *IL36G*) changed after the application of allergen-specific immunotherapy, but only in the case of three genes (*DPP10*, *PLSCR4*, and *IL36G*) did the expression return to the levels observed in the healthy dogs. The expression of the *RARRES2* gene was the lowest in cAD patients prior to immunotherapy, which may be connected to the alternations in retinoid metabolism/signaling in dogs affected by AD. The increased expression of *DPP10* in dogs after 6 months of ASIT in comparison to these patients prior therapy suggests a protective role of *DPP10* in cAD, which is linked with the inhibition of the proinflammatory response through the stimulation of endogenous anti-inflammatory corticosteroid production. In addition, the decreased expression of *DPP10* may be directly connected to the increased levels of *SLPI* transcripts in AD dogs, which, in turn, results in the suppression of TGF-β secretion, causing alterations in the differentiation of Treg cells. Our study also revealed an elevated expression of the *PLSCR4* gene, encoding the phospholipid scramblase membrane proteins that are receptors for SLPI. *PLSCR4* showed the highest expression in cAD patients before therapy, whereas its expression in healthy control dogs and cAD patients after ASIT was similar. The parallel increase in the expression of the *SLPI* and *PLSCR4* transcripts suggests a common pathway of regulation of these genes. Another differentially expressed gene described in our study, *MMP9*, encodes an important matrix metalloproteinase secreted by many inflammatory cells. *MMP9* expression was increased in cAD patients compared to healthy dogs, which might have contributed to the stimulation of the inflammatory processes due to the role of MMP-9 in cleaving the pro-inflammatory chemokines and cytokines secreted by eosinophils, neutrophils, T cells, macrophages, and mast cells. The expression of *MMP9* also remained elevated in dogs subjected to ASIT, suggesting that immunotherapy does not downregulate the levels of *MMP9* transcripts. *NTSR1* was also among the detected genes showing increased expression in AD dogs before ASIT, and lower expression in healthy dogs as well as in cAD patients subjected to therapy. The increased expression of *NTSR1* may be linked with the inflammatory response induced by allergens, due to the fact that this gene encodes neurotensin receptor 1, whose activation results in the secretion of the inflammatory cytokines IL-8 and TNF-α. The genes of β-defensins *CBD103* and *DEFB122* were also among the differentially expressed transcripts detected in our microarray analysis. Beta-defensins show pro- and anti-inflammatory effects, which depend on many factors, such as the disease stage and exposure to pathogens. In our study, the highest expression of *CBD103* was noted in dogs after ASIT and differed significantly from the expression observed in healthy dogs, whereas the expression of *DEFB122* was significantly lower in cAD patients before therapy compared to its expression in healthy dogs and in AD dogs subjected to ASIT. Taken together, this study identifies some novel directions of research on the molecular mechanisms of cAD and immunotherapy used in cAD treatment. Figure 4 illustrates the direct and indirect interactions among the proteins encoded by the differentially expressed genes and cytokines analyzed in the present study. Further research is needed to study the roles of the presented genes and proteins in immune tolerance induction in response to allergen-specific immunotherapy.

## 4. Materials and Methods

### 4.1. Animals

This study complies with national and institutional guidelines on the use of animals in clinical research according to the Polish legal act concerning experiments performed on client-owned animals (Act on Experiments on Animals of 21 January 2005—Journal of Laws of Republic of Poland, abbreviated Dz.U. 2005, No. 33, item 289, as amended). Concerning experiments performed on client-owned animals. All dogs were patients of the Small Animal Clinic at the Warsaw University of Life Sciences. A high standard of care was adhered to throughout each examination. In the case of cAD patients, research was carried out as part of the routine veterinary diagnostic procedure. Dogs included in the control group were blood donors from “Milusia” Veterinary Blood Bank that were submitted to the Small Animal Clinic for routine checkup.

Seven privately owned dogs of various breeds with cAD (five females and two males) were included in this study. The breeds were: Labrador retriever (2), Golden retriever (2), American Staffordshire terrier (2), and small Münsterlander (1). Their ages ranged from 2 years and 2 months to 6.5 years. Eight healthy dogs served as a control group (three females and five males), with their ages ranging between 3 and 8 years. The following breeds were included in the healthy control group: American Staffordshire terrier (2), Labrador retriever (2), bulldog (1), great Dane (1), Staffordshire bull terrier (1), and Weimaraner (1). Detailed information about the dogs included in this study was published in our previous research article [13].

### 4.2. cAD Diagnosis and Sample Collection

The diagnosis of cAD was based on compatible history and clinical signs determined using Willemse and Prélaud diagnostic criteria, followed by Favrot criteria, as follows: pruritus sine materia, an indoor lifestyle, and the exclusion of other causes of pruritus ongoing for at least one year.

In all dogs with chronic pruritus, other causative factors were excluded, i.e., skin parasites (sarcoptic mange, demodectic mange, flea allergic dermatitis). Bacterial pyoderma and Malassezia dermatitis were excluded on the basis of the negative results of in vitro culture assays. The role of food antigens as a cause of the skin condition was assessed using elimination diets for 6–8 weeks. Clinical diagnosis of atopic dermatitis was confirmed via serological allergy testing (IDEXX allergic panel test) and intradermal skin testing (Artuvetrin test set, Lelystad, The Netherlands). No anti-inflammatory drugs were given for at least 3 weeks prior to the serological test and intradermal test.

All dogs classified into the investigated group had positive reactions in serological allergy testing and intradermal skin testing, which was described in our previous article [13]. Peripheral blood samples were collected just before the dogs were subjected to the intradermal skin test, and thus, at the stage when clinical signs of AD were visible, as well as after 3 months and 6 months of therapy. Hematological, morphological, and biochemical blood tests were conducted on samples from qualified patients. Each dog with AD, as well as the animals included in the healthy control group, showed morphological parameters of blood within the reference value range.

The intradermal skin test (Artuvetrin test set, Netherlands) used in the dogs that qualified for the experiment showed that the dogs were primarily sensitized to the storage mites Tyrophagus putrescentiae 86% (6 dogs), Acarus siro 86% (6 dogs), and Lepidoglyphus destructor 57% (4 dogs); the house dust mites Dermatophagoides farine 71% (5 dogs) and Dermatophagoides pteronyssimus 29% (2 dogs); tree pollen mixture 43% (2 dogs); weed pollen mixture 29% (2 dogs); and grass pollen mixture 29% (2 dogs).

### 4.3. Allergen-Specific Immunotherapy (ASIT)

Allergen extracts were prepared on the basis of the results of intradermal tests by the Artuvetrin Therapy company. Allergen extracts were administered subcutaneously in increasing concentrations according to the manufacturer’s recommendations, as described previously [13].

The first dosage for the subcutaneous Artuvetrin Therapy started at 0.2 mL, after which it was gradually increased over longer intervals to a maximum of 1.0 mL. In the 3rd week, the dose was increased to 0.4 mL; in the 5th week, to 0.6 mL; in the 7th, to 0.8 mL; and in 10th, 13th, and 17th, to 1 mL. Then, 1 mL was administered every 4 weeks. In two patients, the time between doses was extended due to hypersensitivity reactions that followed each dose of the allergen extract.

When the 1 mL dose was reached after 12 weeks, a fixed dose of 1.0 mL was administered monthly. In some cases, this treatment schedule was too fast for the patient. If so, it was possible to deviate from the standard dosage schedule.

### 4.4. Gene Expression Microarray Analysis

The blood samples designated for transcriptomic analyses were collected from the dogs twice: before intradermal skin test and immunotherapy, and after six months of ASIT.

Blood samples were collected from AD and healthy dogs into RNeasy Protect Animal Blood Tubes (Qiagen, Germantown, MD, USA). Total RNA from peripheral blood nuclear cells was isolated using an RNeasy Protect Animal Blood Kit (Qiagen, USA). Additionally, contamination with DNA was eliminated via DNAse I digestion, included as an additional step of the isolation protocol. The quantity of RNA was measured using a NanoDrop (NanoDrop Technologies, Wilmington, DE, USA). The analysis of final RNA quality and integrity was performed using an Agilent 2100 Bioanalyzer (Santa Clara, CA, USA) and an RNA 6000 Nano Kit (Agilent, Waldbronn, Germany). To ensure optimal data quality, only RNA samples with RIN number ≥ 7.8 were included in the analysis.

The analysis of gene expression profiles was performed using a Canine (V2) Gene Expression Microarray, 4 × 44 K (Agilent Technologies, USA) and an Agilent Technologies Reagent Set according to the manufacturer’s procedure. An RNA Spike In Kit (Agilent Technologies, USA) was used as an internal control, a Low Input Quick Amp Labeling Kit was applied to amplify and label (Cy3 or Cy5) target RNA to generate complementary RNA (cRNA) for oligo-microarrays, a Gene Expression Hybridization Kit was used for fragmentation and hybridization, and a Gene Expression Wash Buffer Kit was used for washing slides after hybridization. Acquisition and analysis of hybridization intensities were performed using an Agilent DNA Microarray Scanner G2505C. Data were extracted and backgrounds subtracted using the standard procedures included in the Agilent Feature Extraction (FE) Software version 10.7.3.1. FE was used to perform Lowess normalization.

The experiment was performed using a common reference design, in which the common reference comprised a pool of equal amounts of RNA from 13 healthy control dogs. These dogs did not take part in the experiment. The cRNA of the common reference samples was Cy3-labeled, whereas the cRNA of healthy dogs (control group of dogs taking part in experiment) and of cAD patients before ASIT (0) and after 6 months of ASIT (6 months) was labeled with Cy5. Two-color microarrays were performed, one for each patient: 7 microarrays with samples from AD dogs (0), 7 with samples from AD dogs after therapy (6 months), and 8 from healthy control dogs (ctrl). On each microarray, 825 ng of each sample of cRNA (Cy3-labeled common reference and Cy5-labeled control or patient) was hybridized.

To identify signaling pathways and gene function, the microarray data were analyzed using Pathway Studio 12 (Ariadne Genomics, Rockville, MD, USA). This database consisted of millions of individually modeled relationships between proteins, genes, complexes, cells, tissues and diseases.

### 4.5. Real-Time RT-PCR

To verify the microarray results, the expression of four genes (*CBD103*, *IL36G*, *RARRES2*, *SLPI*) was analyzed using real-time qPCR. The sequences of chosen genes were obtained from the Ensembl or NCBI database. Primers were designed using Primer-Blast software (NCBI database) and verified using Oligo Calc: Oligonucleotide Properties Calculator (free software available online, provided by Northwestern University) to exclude sequences showing self-complementarity. To reduce the chances of amplifying traces of genomic DNA, the primers were positioned in different exons. Reference gene *RPS19* was determined using the Genorm and NormFinder programs. All primer sequences are listed in Table 3. Total RNA was reverse transcribed to first-strand complementary DNA (cDNA) using the High-Capacity cDNA Reverse Transcription Kit (Applied Biosystems, USA). All analyses were performed on individual samples of total RNA using SYBR Select Master Mix (Applied Biosystems, USA) on a Stratagene Mx3005P Quantitative PCR instrument for RT-PCR, following the manufacturer’s protocol. For all genes, the annealing temperature was 58 °C. The relative expression of the target gene was quantified as the mean of triplicate measurements for each biological sample. Results were calculated using the 2^−ΔΔCt^ method [75].

### 4.6. Statistical Analysis

The statistical analysis of microarrays was performed using Gene Spring14 software (Agilent, USA). The probe sets were filtered by flags to remove poor quality probes (absent flags); additionally, filtering by error was applied, and outlier data (≥75% of coefficient of variation (CV)) were eliminated.

The statistical significance of the differences observed was evaluated using a one-way ANOVA and Tukey’s HSD post hoc test (*p* < 0.05). Multiple testing correction was performed using Benjamini and Hochberg False Discovery Rate (FDR) < 0.05. Microarray data were deposited in the Gene Expression Omnibus data repository under the number GSE 168109.

The statistical analysis of the real-time qPCR results was performed using GraphPad Prism 7.05 software via one-way ANOVA, in which the mean values of 2^−ΔΔCt^ for samples of patients before ASIT and after 6 months of immunotherapy were compared to the healthy control samples. Statistically significant differences were defined as * *p* < 0.05, ** *p* < 0.01, and *** *p* < 0.001.

## Figures and Tables

**Table 1:** The list of differentially regulated genes in: AD dogs before ASIT vs. dogs after 6 months of ASIT (0 vs. 6 months); AD dogs before ASIT vs. healthy control dogs (0 vs. ctrl); and AD dogs after 6 months of ASIT vs. healthy control dogs (6 months vs. ctrl). The differences in gene expression are presented as fold changes (FCs). The bold FC values are statistically significant (FDR < 0.05), and the “ns” symbol describes changes that are not significant.

Gene Symbol	Gene Name	GenBank Accession	FC (0 vs. 6 Months)	*p*-Values (0 vs. 6 Months)	FC (0 Months vs. Ctrl)	*p*-Values (0 Months vs. Ctrl)	FC (6 Months vs. Ctrl)	*p*-Values (6 Months vs. Ctrl)	Corrected *p*-Values (Ctrl vs. 0 Months vs. 6 Months)
***RARRES2***	*retinoic acid receptor responder 2*	XM_005629654	−1.39 down	ns	**−2.00** **down**	**0.0015**	−1.44 down	ns	**0.0089**
***DPP10***	*dipeptidyl peptidase-like 10*	CX005277	**−1.90** **down**	**0.0006**	**−1.69** **down**	**0.0028**	1.12	ns	**0.0045**
***SLPI***	*secretory leukocyte peptidase inhibitor*	NM_001113171	−1.01	ns	**1.45** **up**	**0.0049**	**1.46** **up**	**0.00426**	**0.0081**
***PLSCR4***	*phospholipid scramblase 4*	XM_005634546	**2.28** **up**	**0.0222**	**2.34** **up**	**0.0144**	1.03	ns	**0.0192**
***MMP9***	*matrix metalloproteinase 9*	NM_001003219	1.08	ns	**3.36** **up**	**0.0239**	**3.10** **up**	**0.0357**	**0.0244**
***NTSR1***	*neurotensin receptor 1*	XM_543088	**2.40** **up**	**0.0014**	**1.67** **up**	**0.0492**	−1.44 down	ns	**0.0083**
***CBD103***	*beta-defensin 103*	NM_001129980	−1.60 down	ns	1.66 up	ns	**2.66** **up**	**0.0240**	**0.0431**
***DEFB122***	*beta-defensin 122*	NM_001024641	**−2.14** **down**	**0.0408**	**−2.88** **down**	**0.0034**	−1.35 down	ns	**0.0123**
***IL36G***	*interleukin 36 gamma*	XM_005630449	**−1.71** **down**	**0.0059**	**−1.99** **down**	**0.0005**	−1.16	ns	**0.0045**

**Table 2:** Functions of differentially expressed genes described in this study.

Gene Symbol	Gene Name	Summary of Gene Function
***RARRES2***	*retinoic acid receptor responder 2*	Encodes a precursor of chemerin that acts via retinoic acid receptor (RAR), causing chemoattraction of dendritic cells and macrophages, linking innate and adaptive immunity [21,22]. *RARRES2* gene is downregulated in skin of AD patients [24].
***DPP10***	*dipeptidyl peptidase-like 10*	Encodes a single-pass type II membrane protein with no detectable protease activity, but with ability to bind other proteins, such as specific voltage-gated potassium channels, altering their expression and biophysical properties. Mutations in this gene have been associated with asthma [25]. Overexpression of *DPP10* enhances glucocorticoid receptor (GR) activation even without glucocorticoid treatment. DPP10 protein may influence endogenous anti-inflammatory corticosteroid production [25].
***SLPI***	*secretory leukocyte peptidase inhibitor*	Encodes a secreted inhibitor protecting epithelial tissues from endogenous serine proteases. SLPI protein also mediates the suppression of TGF-β expression and interferes with the differentiation of Treg cells [28]. SLPI has an affinity for leukocyte elastase and blocks its activity, which is needed for increased TGF-β expression in dendritic cells, in turn, leading to an increased number of CD4 + FOXP3+ cells [28].
***PLSCR4***	*phospholipid scramblase 4*	Encodes a cell membrane protein necessary for CD4 receptor binding activity in T lymphocytes [29]. SLPI binding to PLSCR4 induces its translocation from extracellular matrix to cytoplasm and nuclei of monocytes, macrophages, and B and Th lymphocytes [29].
***MMP9***	*matrix metalloproteinase 9*	Encodes MMP-9—one of the key metalloproteinases involved in the development and course of inflammatory reactions following allergen challenge. MMP-9 is secreted by several inflammatory cells, e.g., eosinophils, neutrophils, T cells, macrophages, and mast cells. MMP-9 promotes migration and activation of immune cells by cleaving pro-inflammatory chemokines and cytokines, therefore contributing to inflammatory processes [30,31,32]. Increased expression of *MMP9* gene is observed in acute compared with chronic AD skin lesions [36].
***NTSR1***	*neurotensin receptor 1*	Encodes a membrane protein belonging to the superfamily of G protein-coupled receptors. NTSR1 is a specific receptor for neurotensin, which, aside from its role as a neurotransmitter, is also known for its pro-inflammatory role, induction of vasodilatation, vascular permeability, activation of mast cell degranulation, and enhancement of directional migration and phagocytosis of neutrophils [43]. Neurotensin can be involved in the pathogenesis of inflammatory skin disorders, including AD, especially those exacerbated by stress, scratching, and sweating [44].
***CBD103***	*beta-defensin 103*	Encodes a protein belonging to defensins—a family of microbicidal and cytotoxic peptides. *CBD103* can be detected in skin and peripheral blood mononuclear cells, but shows decreased expression in cAD patients [56].
***DEFB122***	*beta-defensin 122*	Encodes a protein belonging to defensins—a family of microbicidal and cytotoxic peptides. Decreased expression of *DEFB122* transcript was observed in lesional and non-lesional skin of cAD patients in comparison to the skin of healthy dogs [58].
***IL36G***	*interleukin 36 gamma*	Encodes interleukin 36 gamma (IL-36γ) belonging to the IL-1 superfamily. IL-36 is involved in immune cell activation, antigen presentation, and pro-inflammatory factor production [61]. IL-36 cytokine expression can be found in keratinocytes, B-lymphocytes, T-lymphocytes, dendritic cells, and monocytes [63,64,65,66,67,68]. Increased expression of *IL36G* was shown in lesional skin of AD patients compared to non-lesional skin [67,73,74].

**Table 3:** Primer sequences for real-time qPCR verification of microarray results.

Gene	Forward Primer (5′-3′)	Reverse Primer (5′-3′)	NCBI Accession Number
*CBD103*	TGTGTGTCCTGCAACCTTAT	CACCGACCGCTCCTTATTC	NM 001129980.1
*IL36G*	ATCACTGTTCTCCCATGCAA	CCAGTATCTCCTCCTCCTTTAG	XM_005630449.2
*RARRES2*	GGAGACCAGTGTGGACAGA	CATTTCCGCTTCCTCCCATT	XM_022403838.1
*SLPI*	ATCCCGTTAATGTCTCCAACTC	AATGGCAGGTATCAGGCTTATT	NM_001113171.1
*RSP19*	CCTTCCTCAAAAAGTCTGGG	GTTCTCATCGTAGGGAGCAAG	XM_533657.3

Primers for *SLPI* were designed based on the previously published study by Lancto and coworkers [58], and primers for *RPS19* were designed based on the previous reports by Brinkhof and coworkers [76], Schmitz and coworkers [77], and Majewska and coworkers [20].

## Data Availability

The microarray data presented in this study are openly available in the Gene Expression Omnibus data repository under the number GSE 168109.

## Supplementary Materials

The following supporting information can be downloaded at: https://www.mdpi.com/article/10.3390/ijms241411616/s1.
